# Composed endotypes to guide antibiotic discontinuation in sepsis

**DOI:** 10.1186/s13054-019-2439-0

**Published:** 2019-04-24

**Authors:** Jesus F. Bermejo-Martin, David Andaluz-Ojeda, Marta Martin-Fernandez, Cesar Aldecoa, Raquel Almansa

**Affiliations:** 1 Group for Biomedical Research in Sepsis (BioSepsis), Hospital Clínico Universitario de Valladolid/IECSCYL, Av. Ramón y Cajal, 3, 47003 Valladolid, Spain; 2 Centro de Investigación Biomedica en Red-Enfermedades Respiratorias (CibeRes, CB06/06/0028), Instituto de salud Carlos III (ISCIII), Av. de Monforte de Lemos, 5, 28029 Madrid, Spain; 3 Intensive Care Medicine Service, Hospital Clínico Universitario de Valladolid/IECSCYL, Av. Ramón y Cajal, 3, 47003 Valladolid, Spain; 4 Anesthesiology and Reanimation Service, Hospital Universitario Río Hortega, Calle Dulzaina, 2, 47012 Valladolid, Spain

**Keywords:** Empiric, Antibiotic, Treatment, Sepsis, Severity, Endotypes

## Abstract

Overuse of empiric antibiotic therapy in the ICU is responsible for promoting the dissemination of multidrug-resistant (MDR) bacteria. Shortened antibiotic treatment duration could contribute to palliating the emergence of MDR. Uncertainty about patient evolution is a major concern for deciding to stop antibiotics. Biomarkers could represent a complementary tool to identify those patients for whom antibiotic treatment could be safely discontinued. The biomarker most extensively studied to guide antibiotic withdrawal is procalcitonin (PCT), but its real impact on decreasing the duration of antibiotic treatment is a matter of controversy. Combining biomarkers to rule out complicated outcomes in sepsis patients could represent a better option. Some candidate biomarkers, including mid-regional proadrenomedullin, the percentage of human leukocyte antigen DR (HLA-DR)-positive monocytes, means of fluorescence intensities of HLA-DR on monocytes, interleukin-7 receptor expression levels, immunoglobulin M levels in the serum or the absence of increased proteolysis, have already demonstrated the potential to exclude the risk of progression to septic shock, nosocomial infections, and mortality when tested along the sepsis course. Other promising biomarkers to rule out complicated outcomes are neutrophil protease activity, the adaptive/coagulopathic signatures identified by whole transcriptome analysis by Sweeney et al., and the SRS1 signature identified by Davenport et al. In conclusion, there are a number of promising biomarkers involved in proteolytic, vascular, immunological, and coagulation alterations that could be useful to build composed endotypes to predict uncomplicated outcomes in sepsis. These endotypes could help to identify patients deserving the discontinuation of antibiotics.

Overuse of empiric antibiotic therapy in the ICU is responsible for promoting the dissemination of multidrug-resistant (MDR) bacteria [1]. De-escalation and shortened antibiotic treatment duration are strategies that could contribute to palliating the emergence of MDR [1]. Antibiotic stewardship programs are proposed considering antibiotic cessation in patients exhibiting clinical improvement, but uncertainty about patient evolution is a major concern for deciding to stop antibiotics. Biomarkers could represent a complementary tool to identify patients for whom antibiotic treatment could be safely discontinued. The biomarker most extensively studied to guide antibiotic withdrawal is procalcitonin (PCT). A recent meta-analysis by Lam et al. showed that PCT guidance could help to decrease antibiotic duration and even mortality [2]. In contrast, Pepper et al. concluded that the increased survival and decreased antibiotic utilization associated with PCT-guided antibiotic discontinuation represent low certainty evidence [3].

Relying on a single biomarker is probably not the most appropriate strategy to identify patients deserving antibiotic discontinuation. There are a number of biomarkers that inform the evolution of organ failure and prognosis during sepsis. These biomarkers could be used to build composed endotypes to rule out complicated outcomes, helping to identify the patients qualifying for antibiotic cessation.

## Biomarkers of microcirculatory damage

**Fig. 1:**
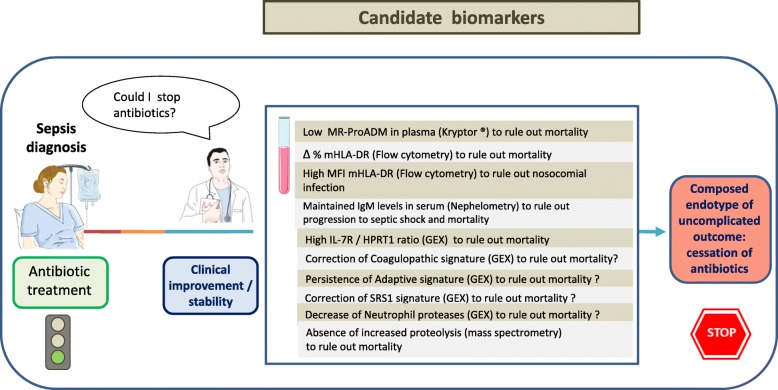
Candidate biomarkers to build composed endotypes to guide antibiotic discontinuation. The presence of an interrogation indicates a biomarker with potential to predict uncomplicated outcome but which at the present moment has been tested just at diagnosis or at ICU admission. Those biomarkers with no interrogation have been tested along the course of sepsis. MR-ProADM: mid-regional proadrenomedullin; mHLA-DR: human leukocyte antigen-DR on monocytes; MFI: means of fluorescence intensities; IL7R: interleukin-7 receptor; HPRT: hypoxanthine phosphoribosyltransferase; SRS1: sepsis response signature; GEX: gene expression. Pictograms were taken from “Smart ServierMedical Art” (https://smart.servier.com/)

Adrenomedullin is a peptide produced by multiple tissues during physiological and infectious stress with varying functions (Fig. 1). Levels of the stable mid-regional fragment of proadrenomedullin (MR-proADM) correlate with organ failure degree in sepsis. Elke et al. showed that low MR-proADM concentrations identify sepsis patients with a very low risk of death throughout the ICU stay [4]. A value of MR-ProADM < 2.25 nmol/L at day 4 or day 7 following ICU admission yielded a negative predictive value (NPV) of 94% to rule out 28-day mortality. In turn, this cutoff yielded an NPV of 96% at day 10 [4].

## Immunological biomarkers

Human leukocyte antigen DR (HLA-DR) represents the capacity of monocytes for antigen presentation and crosstalk to T helper cells, enabling the activation of the adaptive immune system. HLA-DR on monocytes is considered a surrogate marker of sepsis-induced immunosuppression. Survivors show a significant increase in the percentage of HLA-DR-positive monocytes during the course of sepsis [5]. Using flow cytometry in a cohort of patients with severe sepsis, Wu et al. demonstrated that an increment of 4.8% at day 3 following admission to the ICU yielded a NPV of 98% to rule out mortality at 28 days, while an increment of 9% at day 7 yielded an NPV of 97.2% [6]. Landelle et al. also employed flow cytometry and demonstrated that monocyte HLA-DR expressed as the means of fluorescence intensity (MFI) is useful to rule out nosocomial infections after septic shock. Exhibiting > 54 MFIs by days 3–4 following admission to the ICU showed an NPV of 86% to exclude nosocomial infections in these patients [7]. Another promising biological marker to rule out complicated outcomes in sepsis patients during hospitalization in the ICU is interleukin-7 receptor (IL-7R). Interleukin 7 is primarily a survival factor for different subpopulations of lymphoid cells. Using reverse transcription quantitative polymerase chain reaction in whole blood, Delwarde et al. found a gene expression value for IL-7R at day 3 following septic shock diagnosis that yielded a high NPV for 28-day mortality (86%) [8]. This value corresponded to a ratio of 0.20 between the expression of IL-7R and hypoxanthine phosphoribosyltransferase 1 as a reference gene. Immunoglobulin M levels in serum could also help to identify patients with better outcomes. The work of Giamarellos-Bourboulis EJ et al. supported that the kinetics of immunoglobulin M levels in sepsis patients over time could help to rule out progression to septic shock and mortality [9]. Using advanced informatic techniques, Sweeney et al. evidenced the existence in sepsis of a gene expression signature of adaptive immune activation associated with a lower clinical severity and lower mortality [10]. Similarly, Davenport et al. identified a sepsis response signature 1 (SRS1) characterizing individuals with an immunosuppressed phenotype that included features of endotoxin tolerance, T cell exhaustion, and the downregulation of human leucocyte antigen class II, which was associated with higher 14-day mortality [11]. The persistence/presence of the “adaptive” signature or the correction/absence of the SRS1 signature during sepsis could potentially help to identify patients at low risk of developing complications.

## Coagulation and neutrophil protease related biomarkers

Sweeney et al. also found the existence of a gene expression signature of clinical coagulopathy that is associated with higher mortality in sepsis [10]. Evidence of the correction/absence of this signature could also help to identify low-risk patients. Neutrophil proteases are involved in the pathogenesis of the coagulation alterations observed in sepsis and mediate endothelial damage. We have identified that neutrophil protease activity (matrix metallopeptidase 8-MMP8 and lipocalin-2-LCN2/NGAL) is associated with organ failure degree and mortality in sepsis [12, 13]. Evidence of low/decreased levels of neutrophil proteases during hospitalization is also an additional candidate signal to identify uncomplicated patients. Neutrophil proteases mediate proteolysis [14]. Using mass spectrometry, Bauzá-Martinez et al. demonstrated that septic shock survivors are characterized by the absence of increased proteolysis along the evolution of the disease (evidenced by peptide abundance) [14].

## Conclusion

There are a number of promising biomarkers involved in proteolytic, vascular, immunological, and coagulation alterations that could be useful to build composed endotypes predicting uncomplicated outcomes in sepsis. These endotypes could help to identify patients deserving of the discontinuation of antibiotics. Further efforts are needed to identify the optimal biomarker combinations to obtain the best-composed endotype/s identifying patients with uncomplicated outcomes by single or repeated evaluations along the course of the disease. New biomarker profiling technologies, such as automated bedside flow cytometers (Accellix) [15], microfluidic immunoassays (Simple-Plex), and next generation mRNA quantification technologies (droplet digital PCR [13], Nanostring), will facilitate the accurate identification of these composed endotypes.

## Abbreviations

HLA-DR: Human leukocyte antigen-DR
HPRT1: Hypoxanthine phosphoribosyltransferase 1 as reference gene
IL7R: Interleukin-7 Receptor messenger RNA
MFI: Means of fluorescence intensities
MR-ProADM: Mid-regional proadrenomedullin
SRS1: Sepsis response signature 1
